# Australasian interstitial lung disease registry (AILDR): objectives, design and rationale of a bi-national prospective database

**DOI:** 10.1186/s12890-020-01297-2

**Published:** 2020-10-02

**Authors:** Irene Moore, Jeremy Wrobel, Jessica Rhodes, Qi Lin, Susanne Webster, Helen Jo, Lauren Troy, Christopher Grainge, Ian Glaspole, Tamera J. Corte, on behalf of the Australasian ILD Registry, Frank Thien, Ben Kwan, Adelle Jee, Odette Erskine, Alan Teoh, Sally De Boer, Margaret Wilsher, Harry Gallagher

**Affiliations:** 1 Advanced Lung Disease Unit, Fiona Stanley Hospital, PO Box Locked Bag 100, Palmyra DC, Perth, WA 6961 Australia; 2 University of Notre Dame, Fremantle, WA Australia; 3 Royal Prince Alfred Hospital, Sydney, NSW Australia; 4 University of Sydney, Sydney, NSW Australia; 5 John Hunter Hospital, Sydney, NSW Australia; 6 The Alfred Hospital, Melbourne, VIC Australia; 7 Centre of Research Excellence in Pulmonary Fibrosis, Sydney, Australia

**Keywords:** Interstitial lung disease, Lung fibrosis, Autoimmune disease, Clinical epidemiology, Registry

## Abstract

**Background:**

Interstitial Lung Disease (ILD) is a group of respiratory conditions affecting the lung interstitium often associated with progressive respiratory failure. There is increasing recognition of the need for improved epidemiological data to help determine best practice and improve standardisation of care. The Australasian ILD Registry (AILDR) is a bi-national registry of patients with all ILD subtypes designed to establish a clinically meaningful database reflecting real world practice in Australasia with an objective to improve diagnostic and treatment pathways through research and collaboration.

**Methods:**

AILDR is a prospective observational registry recruiting patients attending ILD clinics at centres around Australia and New Zealand. Core and non-core data are stored on a secure server. The pilot phase was launched in 2016 consisting of four sites in Australia. Currently in its second phase a further 16 sites have been recruited, including three in New Zealand.

**Results:**

A total of 1061 participants were consented during the pilot phase. Baseline data demonstrated a mean age 68.3 ± 12.5 (SD) years, mean FVC (%predicted) 79.1 ± 20.4 (SD), mean DLCO (%predicted) 58.5 ± 17.9 (SD) and nadir exertional SpO2 (%) 91 ± 6.9 (SD). Idiopathic pulmonary fibrosis (31%) and connective-tissue disease related ILD (21.7%) were the two most common subtypes. Baseline demographics and physiology were not significantly different across the four centres.

**Conclusion:**

AILDR is an important clinical and research tool providing a platform for epidemiological data that will prove essential in promoting understanding of a rare cohort of lung disease and provide foundations for our aspiration to standardise investigation and treatment pathways of ILD across Australasia.

## Background

Interstitial lung disease (ILD) encompasses a heterogeneous group of respiratory disorders characterised by inflammation and/or fibrosis of the lung interstitium. Broadly speaking, ILD can be divided into four main groups [1]. Firstly, there are the Idiopathic Interstitial Pneumonias (IIPs) including Idiopathic Pulmonary Fibrosis (IPF), the most common IIP, along with idiopathic non-specific idiopathic pneumonia (iNSIP), acute interstitial pneumonia (AIP) and respiratory bronchiolitis-associated ILD (RB-ILD), to name a few. ILD attributable to known causes such as connective tissue disease (CTD-ILD) or specific exposures; granulomatous ILD including sarcoidosis and hypersensitivity pneumonitis (HP); and rare forms of ILD such as lymphangioleiomyomatosis (LAM) or Langerhans cell histiocytosis (LCH) account for the remaining subgroups.

ILD includes a spectrum of clinical phenotypes. Delineating the specific ILD pattern and disease behaviour is now, more than ever, pertinent to management. Morbidity and mortality, as well as treatment options differ between subtypes. For example, the use of anti-fibrotic agents and avoidance of immunosuppression is paramount in IPF comparative to CTD-ILD where immunosuppression is often first line therapy [2–5]. There is now also increasing recognition of the “progressive fibrotic” phenotype across disease subtypes, with recent publications highlighting a potential role for anti-fibrotics in these conditions in addition to standard therapy [6, 7]. To establish accurate diagnoses, guidelines mandate thorough clinical history and examination combined with high resolution CT imaging and autoimmune serology. These data should be presented to an ILD multi-disciplinary meeting (MDM) with consideration of lung biopsy in cases with persisting diagnostic uncertainty [8]. Discussing cases at an ILD MDM with sufficient subspecialty expertise can significantly improve diagnostic accuracy [9].

There is now increasing momentum calling for improved epidemiological data. Whilst the availability of incidence and prevalence data in IPF has greatly improved over the years, little information is available for other ILDs [10]. A number of ILD advocacy groups have highlighted the need worldwide for ILD registries to provide critical real world data, aspiring to translate this knowledge into improved clinical care and patient outcomes [11]. Further rationale for this is highlighted by the lack of data to inform standardised diagnostic and treatment approaches, particularly for the rarer ILD subgroups.

A national (or international) ILD registry offers an opportunity to understand disease patterns, standardise care and provide relevant longitudinal data. The Australian IPF Registry (AIPFR) has been recruiting patients successfully since 2012. This internationally acclaimed registry has 817 participants recruited, to August 2019. Following the success in working across multiple centres in this nationally coordinated registry, we launched the Australasian ILD Registry (AILDR) inclusive of all ILD diagnoses in centres across Australia and New Zealand.

### The Australasian ILD Registry overview

**Fig. 1:**
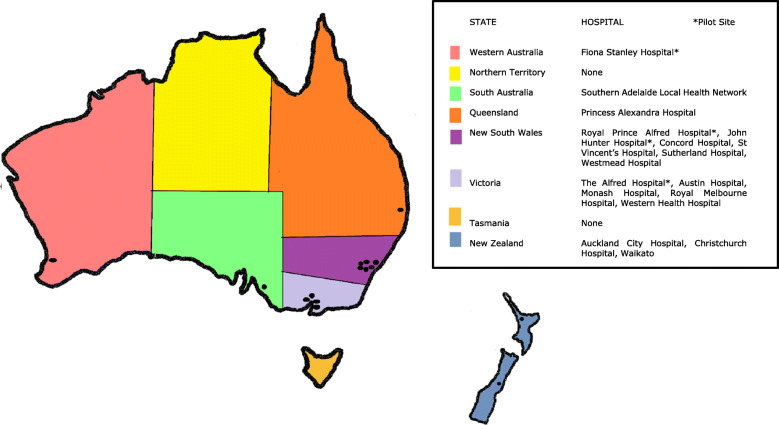
Map of Australia and New Zealand with participating ILD registry recruiting centres (marked by black dot) including the four pilot sites (Royal Prince Alfred, John Hunter, Alfred, Fiona Stanley)

AILDR is a bi-national prospective observational cohort registry designed to recruit patients attending ILD clinics at both tertiary and general centres around Australia and New Zealand (see Fig. 1.). All citizens with any form of ILD are eligible for recruitment unless rejected on exclusion criteria. The registry was launched in three anticipated phases; a pilot study of four sites, a second phase to recruit a further 16 sites and the third phase to ensure ongoing prospective data collection and recruitment of both patients and additional centres. The four site pilot study is now complete (2016–2018) and the second phase of recruitment is underway. Ethical approval for the registry was granted by the Sydney Local Health District HREC on 1st September 2016 (HREC/16/RPAH/345) and the Western Australian South Metropolitan Health Service HREC on 5th May 2017 (RGS11/ILD1) with each site responsible for obtaining local governance approval.

The objectives of AILDR are to 1) establish the incidence and prevalence of ILD subtypes across Australasia; 2) to provide a clinically meaningful database to facilitate quality improvement (e.g. establishing bi-national diagnostic and treatment pathways); 3) to provide data on real world treatment practice; and 4) to enable collaborative research particularly of rare forms of ILD. Inclusion criteria are patients > 18 years of age, able to give informed consent and with a diagnosis of ILD, where applicable, according to American Thoracic Society/European Respiratory Society (ATS/ERS) criteria. Exclusion criteria are those < 18 years of age or those unable to give informed consent. All patients are provided with both verbal and written information and advised they can withdraw consent at any time, without affect ongoing clinical care. AILDR is designed as an opt-in registry therefore any centre with an ILD MDM that wishes to join is welcomed.

Lung Foundation Australia (LFA) provides registry governance, serving to support the purpose and strategic goals of the AILDR, provide oversight of the agreed protocols with appropriate ethics, provide qualified personnel and ensure that deliverable measures are in place. Individual sites have signed a memorandum of understanding with LFA prior to recruitment. A registry steering committee convenes quarterly with attendance expected from the Principle Investigator (PI) at each site.

## Methods

For all participants, retrospective data is entered after consent at first clinic visit with prospective data entered after each subsequent clinic visit. Data is recorded on a secure server hosted by a leading international server hosting infrastructure company using third party database software FileMaker (initially version Pro15, subsequently updated to Pro17). Responsibility for data entry falls to the PI or nominated co-investigator(s) at each site. There is a project manager with overall access to the registry but only local individual data can be accessed by each site. No additional visits or investigations are performed for the sole purpose of the registry and the frequency of objective testing and clinic review is determined independently by each site.

A summary of core data recorded on the registry is demonstrated in Table 1. This includes basic demographic data such as sex, age and ethnicity. Clinical data includes detailed descriptions of presenting symptoms, clinical findings, occupational and environmental exposures, family history and co-morbid disease. Details about the first onset of symptoms with cross reference to the diagnosis date was used to capture incidence and prevalence rates. Current and past medication lists are recorded including oxygen use. ILD diagnosis is chosen from a pre-specified drop-down list of diagnoses, reflecting the local ILD MDM consensus findings. Results of investigations performed as part of baseline and ongoing assessment are recorded, including serum blood markers, high resolution CT chest findings, blood gases, bronchoscopy +/− biopsy. Functional parameters include standardised pulmonary function tests (PFTs), and 6-min walk test (6MWT). Participants are treated according to clinical practice at each site. Active and past treatments specific to any form of ILD are encouraged to be recorded, as is reporting of any adverse effects or incidents, although neither are mandatory. Mortality data is reviewed every 6 months with dates of death and lung transplantation recorded as determined by clinical records and/or death certificates.

**Table 1:**
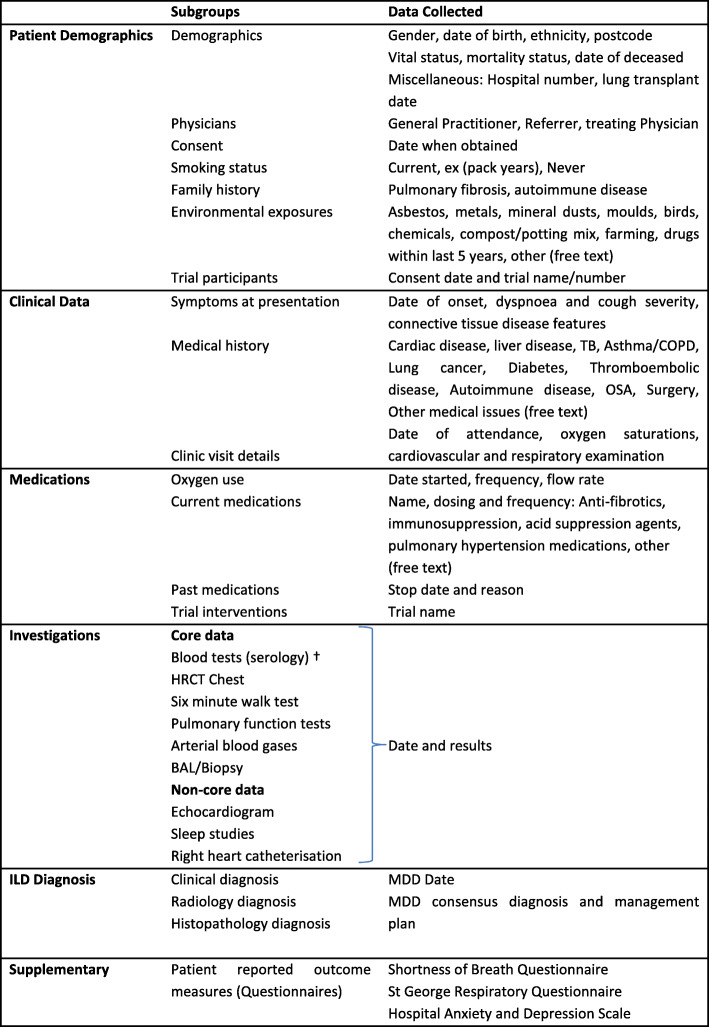
AILDR registry data headings including information collected

*Abbreviations*: *ILD* Interstitial lung disease, *TB* Tuberculosis, *COPD* Chronic obstructive pulmonary disease, *OSA* Obstructive sleep apnoea, *BAL* Bronchoalveolar lavage, *HRCT* High resolution computerised tomography, *MDD* multi-disciplinary discussion

^a^Blood test list available as supplement

Supplementary (or non-core) data includes tests such as echocardiogram, sleep studies and right heart catheterisation and is recorded at investigators’ discretion. Sites are also authorised to record any of the following approved questionnaires which were performed; Shortness of Breath Questionnaire (SOBQ- Australia/English Version 2011), St George Respiratory Questionnaire (SGRQ – UK/English original version) and Hospital Anxiety and Depression Scale (HADS – undated).

Inbuilt quality control functions exist within the registry database – alerts for out of range for example. Additionally, a data manager facilitates ‘cleaning data’ with regular checks. *Results.*

Baseline data of the AILDR registry pilot phase is summarised in Table 2. The pilot phase consisted of four sites and were chosen on merit for having pre-existing ILD structured clinics and MDMs with dedicated ILD leads experienced in research; Royal Prince Alfred Hospital NSW, John Hunter Hospital NSW, The Alfred Hospital VIC and Fiona Stanley Hospital WA. A total of 1061 patients were recruited during the pilot phase: RPA *n* = 511 (48.2%), JHH *n* = 204 (19.2%), TAH *n* = 158 (14.9%) and FSH *n* = 188 (17.7%).

**Table 2:** Baseline demographic and physiological data of registry pilot phase (4 sites)

Variable	All sites	Royal Prince Alfred Hospital	John Hunter Hospital	Fiona Stanley Hospital	The Alfred Hospital
Number of participants	1061	511	204	188	158
Mean age, years (SD)	68.3 (±12.5)	67.9 (±12.9)	73.9 (±10)	64.9 (±12.7)	66.5 (±11.4)
Male (% total)	532 (54.7)	287 (56.4)	96 (47.1)	46 (44.6)	103 (65.6)
Mean FVC, % predicted (SD) ^a^	79.1 (±20.4)	77.7 (±19.8)	91.4 (±21.3)	82.9 (±23.9)	81.2 (±22.9)
Mean DLCO, % predicted (SD) ^a^	58.5 (±17.9)	60.8 (±17.8)	52.5 (±16.3)	58.7 (±22.9)	59 (±21.5)
Mean 6MWT distance, metres (SD) ^a^	456.3 (±120.7)	438.8 (±127.5)	394.6 (±83.2)	432.9 (±125.9)	444.4 (±124.5)
Mean 6MWT nadir SpO2, % (SD) ^a^	91.2 (±6.9)	91.8 (±7.3)	86.2 (±6.2)	89.8 (±6.3)	86.3 (±6.4)

*Abbreviations*: *SD* Standard deviation, *FVC* Forced vital capacity, *DLCO* Diffusing capacity for carbon monoxide, *6MWT* six minute walk test, *SpO2* oxygen saturations

^a^Percentages calculated on non-missing data

The mean age of participants was 68.3 years (±12.5 SD) of whom 54.7% were male. Mild to moderate restrictive defects were observed on pulmonary function testing with a mean FVC (%predicted) 79.1 (±20.4 SD) and mean DLCO (%predicted) 58.5 (±17.9 SD). The mean 6MWT distance (metres) was 456.3 (±120.7 SD) and nadir SpO2 (%) 91 (±6.9 SD). A total of 150 participants completed questionnaires. ILD diagnoses is summarised in Table 3. In the pilot phase 31% had a diagnosis of IPF with CTD-ILD accounting for 21.7%. Those with a subsequent change in ILD diagnosis to non-ILD were not removed from the registry but had no subsequent data recorded. Baseline demographics, physiology and ILD diagnoses were not significantly different across the four centres.

**Table 3:** Recorded ILD diagnoses in completed data sets (*n* = 705) up to 1st August 2019

ILD classification	ILD diagnosis	Total number of patients to 1st August 19
Idiopathic Interstitial	Idiopathic Pulmonary Fibrosis (IPF)	240 (34%)
Pneumonias (IIP)	Non-specific interstitial pneumonia (iNSIP)	29 (4.1%)
	Desquamative Interstitial Pneumonia (DIP)	2 (0.3%)
	Combined Pulmonary Fibrosis and Emphysema (CPFE)	30 (4.3%)
	Cryptogenic Organising Pneumonia (COP)	14 (2%)
	Lymphocytic Interstitial Pneumonia (LIP)	2 (0.3%)
	Respiratory Bronchiolitis Associated ILD (RB-ILD)	9 (1.3%)
	Acute interstitial pneumonia (AIP)	1 (0.1%)
	Unclassifiable^a^	51 (7.2%)
ILD of known association	Connective Tissue Disease associated ILD (CTD-ILD)	125 (17.7%)
	Drug induced ILD	7 (1.0%)
	Occupational exposures	11 (1.6%)
Granulomatous ILD	Hypersensitivity Pneumonitis (HP)	66 (9.4%)
	Sarcoidosis	44 (6.2%)
	Vasculitis associated ILD	12 (1.7%)
Miscellaneous ILD	Lymphangioleiomyomatosis (LAM)	2 (0.3%)
	Langerhan’s cell histiocytosis (LCH)	1 (0.1%)
Other	Early ILD – Interstitial Lung Abnormality	5 (0.7%)
	Interstitial Pneumonia with Autoimmune features (IPAF)	3 (0.4%)
	Pulmonary Alveolar Proteinosis	1 (0.1%)
	Not ILD^b^	18 (2.6%)
	Not specified	32 (4.8%)

^a^Defined as < 50% diagnostic certainty of any diagnosis (Ryerson Classification)

^b^Includes patients initially managed as ILD with subsequent change in diagnosis

**Fig. 2:**
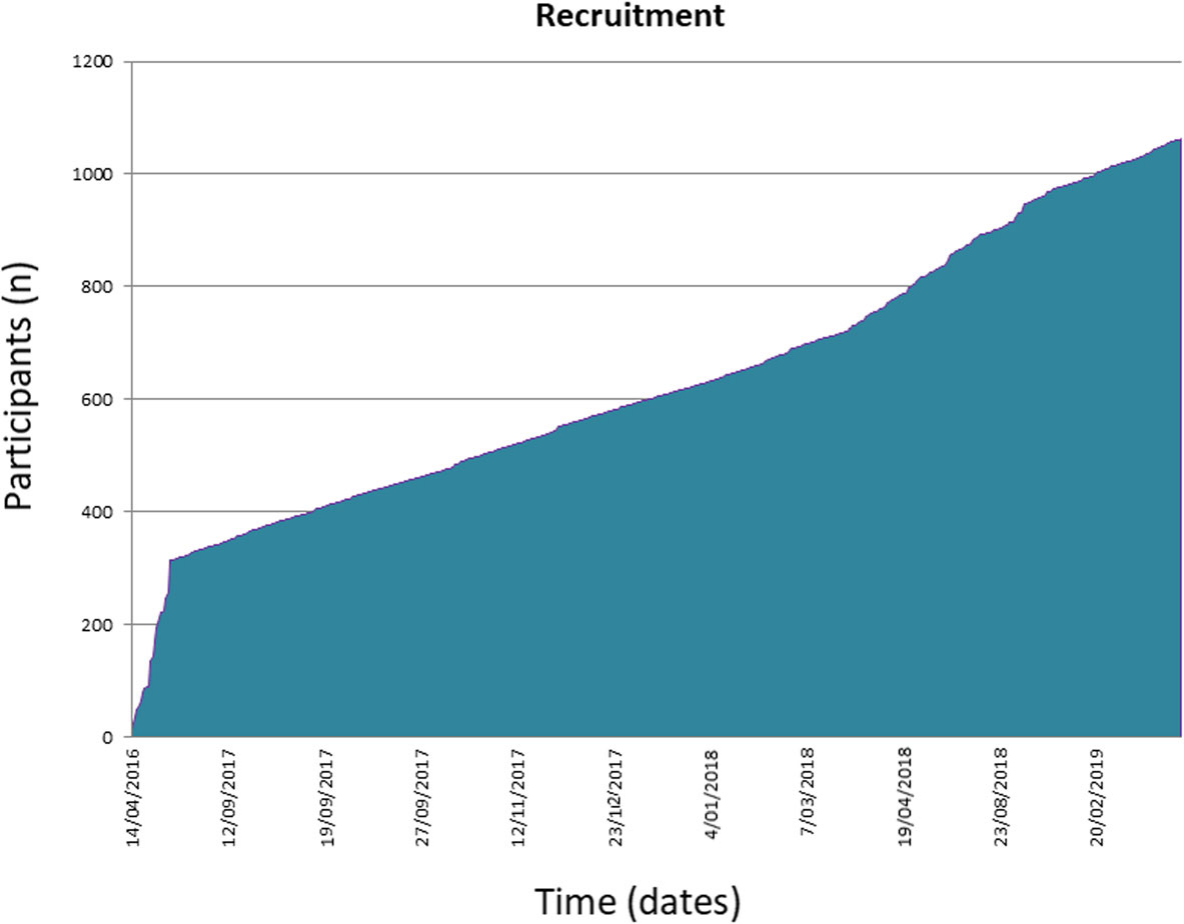
Graph demonstrating recruitment to AILDR from initiation in 2016 to August 2019

Phase two is ongoing and as of 1st August 2019 has 1312 participants (705 completed data sets) in 20 sites across Australia and New Zealand (Fig. 2.). In this bigger cohort, 34% have IPF and 17.8% have CTD-ILD.

## Discussion

Establishing an ILD bi-national registry is of paramount importance in developing services and treatment for a cohort of patients with diseases that still require significant clinical understanding. A meaningful clinical and research database such as AILDR has the potential to identify predictors of outcome aiding the physician when considering escalation of care or referring for transplant. Comparatively, much work has been dedicated to establish IPF registries globally and facilitated several large multinational placebo-controlled trials [12]. Prior to this clinical practice in IPF was derived from single centre observational studies [13]. For non-IPF ILD, similar platforms must now be facilitated, recognising the morbidity and mortality associated with these often neglected diseases.

A number of national ILD registries have emerged in recent years and are discussed in detail elsewhere. Direct comparison between these registries is understandably difficult with many specific to IPF only or, unlike AILDR, inclusive of some, but not all ILD. Furthermore, there is no internationally accepted agreement on what constitutes core and non-core data. However, we show in our pilot phase, that our baseline demographics and physiology is somewhat similar to other reported data, particularly from the European registries, Table 4.

**Table 4:** Table of national ILD registries (excluding IPF only registries)

Country	Registry Name (www.clinicaltrials.gov Identifier)^a^	Data Collection	Population (and size)	Mean FVC, % predicted (SD or range)	Mean DLCO (%predicted)
Australia and New Zealand	AILDR	May 2016 - current	ILDs inc. IPF (> 1300)	79.1 (±20.4)	58.5 (±17.9)
Canada	CARE-PF [14]	2016 - current	Fibrotic ILDs inc. IPF (> 3000)	74.5 (±20.3)	56.7 (±20.1)
United States	PFF-PR [15] (NCT02758808)	Aug 2018 - current	ILDs inc. IPF (> 1400)	68 (±20)	45 (±18)
Germany	EXCITING registry [16] (NCT02645968)	Oct 2014 - current	ILDs inc. IPF (> 200)	72	51
Romania	REGIS [17]	2014–2017	ILDs inc. IPF (> 100)	94.1	78.1
Turkey	TURK-UIP (NCT02821039)	June 2016 – July 2019	ILDs with UIP (> 1600)	Not published	Not published
India	ILD-India [18]	March 2012 – June 2015	ILDs inc. IPF (> 1000)	57.2 (±23.3)	45.4 (±41.6)
Japan	JIIPS Registry [19] (NCT03041623)	Dec 2016 – March 2021	ILDs inc. IPF (> 860)	82 (69.1–93.9)	67.1 (53.5–83)
Italy	RIPID [20]	1997–2005	ILDs (> 3100)	Not published	Not published
Greece	[21]	Jan 2004 – Dec 2004	ILDs inc. IPF (> 960)	Not published	Not published
Seoul	Interstitial Lung Disease Registry Construction (NCT03238989)	Jan 2014 – Dec 2023	ILDs inc. IPF (Est. 300)	Not published	Not published
New Mexico	New Mexico Interstitial Lung Disease Registry [22]	Oct 1988-Sept 1990	ILDs inc. IPF (> 450)	69.1 (±21.6)	Not published
Saudi Arabia	[23]	2008–2011	ILDs inc. IPF (> 300)	66.1(±20.8)	44.4 (±19.5)
Belgium (Flanders)	[24]	1992–1996	ILDs inc. IPF (> 360)	82 (±22)	77 (±19)
Denmark	[25]	Apr 2003 – Mar 2009	ILDs inc. IPF (> 430)	71.3 (±22.2)	48.5 (±19.0)
Denmark	DANILDA	Jan 2018 – current	ILDs inc. IPF (> 250)	Not published	Not published
Spain	RENIA [26]	1998–2000	ILDs inc. IPF (> 740)	Not published	Not published
United States	IPF-PRO/ILD-PRO Registry (NCT01915511)	June 2016 - current	Progressive ILDs inc. IPF (est. 2000)	Not published	Not published
United States	RAPID (NCT03297775)	June 2017 - current	RA and ILD inc. IPF (Est. 500)	Not published	Not published
International	EUSTAR [27]	June 2004 - current	SSc inc. SSc-ILD (> 15,000)	92.2 (±21.3)	68.3 (±21.1)
Australia	SASR [28]	1993–2007	Scleroderma (786)	Not published	Not published
UK	BRILL [29]	1987 - current	RA and ILD (230)	101 (54–145)^b^ 70 (44–117)^c^	61 (33–106)^b^ 52 (22–109)^c^
UK	BTS [30]	Feb 2013 - current	Sarcoidosis (> 300)	97	78.2
Europe	eurIPFreg (NCT02951416)	Sept 2009 - current	ILDs inc. IPF (> 1080)	Not published	Not published

*Abbreviations*: *AILDR* Australasian ILD registry, *CARE-PF* The Canadian Registry for Pulmonary Fibrosis, *PFF-PR* Pulmonary Fibrosis Foundation Patient Registry, *REGIS* Romanian Registry for Interstitial Lung Diseases, *UIP* Usual Interstitial Pneumonia, *RIPID* Registry of Diffuse Infiltrative Pulmonary Diseases, *RAPID* Rheumatoid Arthritis patients at Risk for ILD, *RA* Rheumatoid Arthritis, *EUSTAR* European Scleroderma Trials and Research Group, *SSc* Systemic Sclerosis, *SASR* South Australia Scleroderma Register, *BRILL* British Rheumatoid Interstitial Lung network, *BTS* British Thoracic Society

^a^Trial identifier where available

^b^Limited disease on CT

^c^Extensive disease on CT

The potential benefits of the AILDR are significant. Having access to large numbers of patients with relatively rare disease facilitates audits of practice, disease trends and predictors of prognosis, identification of patients for clinical trials and other research platforms, and encourages collaboration among ILD centres to promote standardisation of care specific to Australasia. It is important to acknowledge that data collected as part of AILDR is real world, non-randomised data and therefore determining causal association is not feasible but this should not negate the invaluable information it provides. Incorporating relevant data into clinical practice, be it prognostication, determining objective testing timeframes, or developing a bi-national diagnostic pathway will ultimately best serve our patients. It can enable accurate health cost benefit analysis and future planning, the latter point particularly prudent in an era of population aging and increased use of expensive ILD specific drugs.

Lessons learned from the AILDR pilot study have prompted the need for clear enunciation of our objectives, guidance for mandatory versus non-mandatory data fields and recognition of future funding requirements for personnel and overheads to maintain the registry. Additionally, focus on establishing a collated network of physicians, patient advocate groups and potential sponsors is essential to continue momentum and ensure registry longevity.

There are of course several factors to overcome with establishing any registry and particularly one on a bi-national scale [31]. Initiating a large, multi-centred registry requires enthusiasm from individual centres, appointed personnel with dedicated time to collate and upload data and local infrastructure with dedicated ILD clinics and expertise. The topographical nature of both countries, but particularly Australia, means there are vast distances between towns and cities limiting physical access to clinics with potential for missed cases. In that regard there has been a substantial push for ‘telehealth’ applications here in Australia to facilitate virtual attendance at clinics aiding our objective of defining ILD across Australasia. Determining sites with sufficient ILD expertise is also challenging and having a central unit who reviews all submissions including diagnosis accuracy would be desirable. Although there is potential for diagnostic variability across centres, this reflects real world practice and broadens the applicability of findings. The registry is reliant on the insertion of accurate and consistent data in a timely manner so maintaining momentum and motivation is paramount and may prove challenging. There is often difficulty balancing clinical versus research needs and thus the registry must function as a useful adjunct to clinical practice for participating clinicians to respond positively.

It would be remiss not to acknowledge some selection bias within our registry population. It is impossible to know what percentage of patients are managed in specialist clinics versus community respiratory or medical physician led clinics and thus we appreciate there is likely to be some selection bias in those that attend ILD centres. As in any registry based study, it may be that simpler, stable patients are not referred to specialist clinics as often thus effecting reporting of true incidence and prevalence rates. Consequently we have addressed this by inviting all tertiary and smaller community hospitals with an ILD MDM to participate.

Additional barriers specific to AILDR included obtaining bi-national ethical approvals, lengthy local governance processes, agreements on funding, establishing proxy server access and general variation in both interstate and international practices. Considerable costs are associated with the upkeep of the secure server on which data is stored, the funding of a designated research officer or project manager and perhaps, in the future, biospecimen procurement. Importantly, whilst barriers to AILDR have been discussed, strong leadership and enthusiasm for a much needed resource continues to move the registry forward.

## Conclusion

The AILDR has been tasked to establish the first Australasian research platform for much needed epidemiological data on the spectrum of ILD. It follows the success of the AIPFR with many of the key stakeholders involved in that project now on the steering committee of this present registry. AILDR aims to facilitate collaborative research, identify factors predictive of prognosis and treatment response, and to provide insight into rarer forms of ILD. Despite challenges the registry continues to thrive. Ongoing success will rely on commitment to accurate diagnoses, submission of clinical data and recurrent funding. This initiative paves the way for global collaboration of much needed research in this ever- evolving field of respiratory medicine.

## Supplementary information

**Additional file 1.**

## Data Availability

The datasets supporting the conclusions of this article are available on request through the Registry co-ordinators (Jessica Rhodes: Jessica.Rhodes@health.nsw.gov.au, Qi ‘Tina’ Lin: Qi.Lin@health.nsw.gov.au). Restrictions apply to the availability of these data with requests to be approved by the Steering Committee prior to access.

## Abbreviations

AIP: Acute interstitial pneumonia
AILDR: Australasian ILD Registry
AIPFR: Australian IPF Registry
CT: Computerised tomography
CTD-ILD: Connective tissue disease associated ILD
DLCO: Diffusing capacity for carbon monoxide
FVC: Forced vital capacity
HADS: Hospital Anxiety and Depression Scale
HP: Hypersensitivity pneumonitis
IIP: Idiopathic Interstitial Pneumonias
ILD: Interstitial lung disease
IPF: Idiopathic Pulmonary Fibrosis
LAM: Lymphangioleiomyomatosis
LCH: Langerhans cell histiocytosis
LFA: Lung Foundation Australia
MDM: Multi-disciplinary meeting
NSIP: Non-specific idiopathic pneumonia
PI: Principle Investigator
PFTs: Pulmonary function tests
RB-ILD: Respiratory bronchiolitis-associated ILD
SGOQ: St George Respiratory Questionnaire
SOBQ: Shortness of Breath Questionnaire
Sp02: Oxygen saturations
6MWT: 6-min walk test

## References

[1] Travis WD, Costabel U, Hansell DM, King TE, Lynch DA, Nicholson AG (2013). An official American Thoracic Society/European Respiratory Society statement: update of the international multidisciplinary classification of the idiopathic interstitial pneumonias. Am J Respir Crit Care Med.

[2] Richeldi L, du Bois RM, Raghu G, Azuma A, Brown KK, Costabel U (2014). Efficacy and safety of nintedanib in idiopathic pulmonary fibrosis. N Engl J Med.

[3] Noble PW, Albera C, Bradford WZ, Costabel U, Glassberg MK, Kardatzke D (2011). Pirfenidone in patients with idiopathic pulmonary fibrosis (CAPACITY): two randomised trials. Lancet.

[4] The Idiopathic Pulmonary Fibrosis Clinical Research Network (2012). Prednisone, azathioprine, and N-Acetylcysteine for pulmonary fibrosis. N Engl J Med.

[5] Volkmann ER, Tashkin DP, Sim M, Li N, Khanna D, Roth MD (2019). Cyclophosphamide for systemic sclerosis-related interstitial lung disease: a comparison of scleroderma lung study I and II. J Rheumatol.

[6] Distler O, Highland KB, Gahlemann M, Azuma A, Fischer A, Mayes MD (2019). Nintedanib for systemic sclerosis–associated interstitial lung disease. N Engl J Med.

[7] Flaherty KR, Wells AU, Cottin V, Devaraj A, Walsh SLF, Inoue Y (2019). Nintedanib in progressive fibrosing interstitial lung diseases. N Engl J Med.

[8] Raghu G, Remy-Jardin M, Myers JL, Richeldi L, Ryerson CJ, Lederer DJ (2018). Diagnosis of idiopathic pulmonary fibrosis. an official ATS/ERS/JRS/ALAT clinical practice guideline. Am J Respir Crit Care Med.

[9] Cottin V, Castillo D, Poletti V, Kreuter M, Corte TJ, Spagnolo P (2018). Should patients with interstitial lung disease be seen by experts?. Chest..

[10] Olson AL, Gifford AH, Inase N, Fernández Pérez ER, Suda T (2018). The epidemiology of idiopathic pulmonary fibrosis and interstitial lung diseases at risk of a progressive-fibrosing phenotype. Eur Respir Rev.

[11] Farrand E, Anstrom KJ, Bernard G, Butte AJ, Iribarren C, Ley B (2019). Closing the evidence gap in interstitial lung disease. The promise of real-world data. Am J Respir Crit Care Med.

[12] Cottin V, Maher T (2015). Long-term clinical and real-world experience with pirfenidone in the treatment of idiopathic pulmonary fibrosis. Eur Respir Rev.

[13] Raghu G (2017). Idiopathic pulmonary fibrosis: lessons from clinical trials over the past 25 years. Eur Respir J.

[14] Fisher JH, Kolb M, Algamdi M (2019). Baseline characteristics and co-morbidities in the CAndian REgistry for pulmonary fibrosis. BMC Pulm Med.

[15] Flaherty K, De Andrade J, Lancaster L (2018). Baseline characteristics of 1461 participants in the Pulmonary Fibrosis Foundation Patient Registry [abstract]. Eur Respir J.

[16] Kreuter M, Herth FJF, Wacker M (2018). Interims analysis of the EXCITING-ILD registry (registry for exploring clinical and epidemiological characteristics of interstitial lung diseases)[abstract]. Eur Respir J.

[17] Strambu I, Salmen T, Traila D (2017). Romanian Registry for Interstitial Lung Diseases (REGIS): inclusion of patients in 3 years [abstract]. Eur Respir J.

[18] Singh S, Collinf BF, Sharma BB (2017). Interstitial lung disease in India: results of a prospective registry. Am J Respir Crit Care Med.

[19] Fujisawa T, Mori K, Mikamo M (2019). Nationwide cloud-based integrated database of idiopathic interstitial pneumonias for multidisciplinary discussion. Eur Respir J.

[20] Tinelli C, De Silvestri A, Richeldi L, Oggionni T (2005). The Italian register for diffuse infiltrative lung disorders (RIPID): a four-year report. Sarcoidosis Vasc Diffuse Lung Dis.

[21] Karakatsani A, Papakosta D, Rapti A (2009). Epidemiology of interstitial lung diseases in Greece [abstract]. Respir Med.

[22] Coultas DB, Zumwalt RE, Black WC, Sobonya RE (1994). The epidemiology of interstitial lung diseases. Am J Respir Crit Care Med.

[23] Alhamad EH (2013). Interstitial lung diseases in Saudi Arabia: a single-center study. Ann Thorac Med.

[24] Thomeer M, Demedts M, Vandeurzen K (2001). Registration of interstitial lung diseases by 20 centres of respiratory medicine in Flanders. Acta Clin Belg.

[25] Hyldgaard C (2015). A cohort study of Danish patients with interstitial lung diseases. lung diseases: Burden, severity, treatment and survival [abstract]. Dan Med J.

[26] López-Campos JL, Rodríguez-Becerra E, Neumosur Task Group (2004). Incidence of interstitial lung diseases in the south of Spain 1998-2000: the RENIA study. Eur J Epidemiol.

[27] Meier FMP, Frommer KW, Dinser R (2012). Update on the profile of the EUSTAR cohort: an analysis of the EULAR scleroderma trials and research group database. Ann Rheum Dis.

[28] Hissaria P, Lester S, Hakendorf P, Woodman R, Patterson K, Hill C, Ahern MJ, Smith MD, Walker JG, Roberts-Thomson PJ (2011). Survival in scleroderma: results from the population-based South Australian Register. Intern Med J.

[29] Kelly CA, Saravanan V, Nisar M, Arthanari S, Woodhead FA, Price-Forbes AN, Dawson J, Sathi N, Ahmad Y, Koduri G, Young A, on behalf of the British Rheumatoid Interstitial Lung (BRILL) Network (2014). Rheumatoid arthritis-related interstitial lung disease: associations, prognostic factors and physiological and radiological characteristics—a large multicentre UK study. Rheumatology.

[30] Thillai M, Chang W, Chaudhuri N, on behalf of the British Thoracic Society (2019). Sarcoidosis in the UK: insights from British Thoracic Society registry data. BMJ Open Respi Res.

[31] Culver DA, Behr J, Belperio JA, Corte TJ, de Andrade JA, Flaherty KR (2019). Patient registries in idiopathic pulmonary fibrosis (IPF). Am J Respir Crit Care Med.

