# Supplementary figures and images for: Australasian interstitial lung disease registry (AILDR): objectives, design and rationale of a bi-national prospective database
Source: BMC Pulm Med. 2020 Oct 2;20:257. doi: 10.1186/s12890-020-01297-2 (PMC7532571; doi:10.1186/s12890-020-01297-2)

Supplement 1. List of ILD blood screening tests


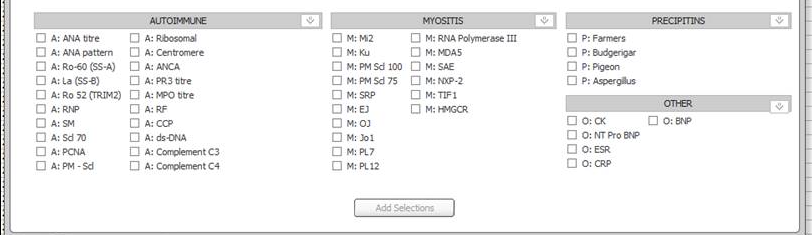

Supplement: Supplementary file 1 — Additional file 1. [file 12890_2020_1297_MOESM1_ESM.docx]
